# High-Throughput Prediction and Design of Novel Conopeptides for Biomedical Research and Development

**DOI:** 10.34133/2022/9895270

**Published:** 2022-11-07

**Authors:** Bingmiao Gao, Yu Huang, Chao Peng, Bo Lin, Yanling Liao, Chao Bian, Jiaan Yang, Qiong Shi

**Affiliations:** ^1^Key Laboratory of Tropical Translational Medicine of Ministry of Education, School of Pharmacy, Hainan Medical University, Haikou, Hainan 570102, China; ^2^Shenzhen Key Lab of Marine Genomics, Guangdong Provincial Key Lab of Molecular Breeding in Marine Economic Animals, BGI Academy of Marine Sciences, BGI Marine, Shenzhen, Guangdong 518081, China; ^3^BGI-Marine Research Institute for Biomedical Technology, Shenzhen Huahong Marine Biomedicine Co. Ltd., Shenzhen, Guangdong 518119, China; ^4^Hainan Provincial Key Laboratory of Carcinogenesis and Intervention, Hainan Medical University, Haikou, Hainan 570102, China; ^5^Research and Development Department, Micro Pharmtech Ltd., Wuhan, Hubei 430075, China

## Abstract

Cone snail venoms have been considered a valuable treasure for international scientists and businessmen, mainly due to their pharmacological applications in development of marine drugs for treatment of various human diseases. To date, around 800 *Conus* species are recorded, and each of them produces over 1,000 venom peptides (termed as conopeptides or conotoxins). This reflects the high diversity and complexity of cone snails, although most of their venoms are still uncharacterized. Advanced multiomics (such as genomics, transcriptomics, and proteomics) approaches have been recently developed to mine diverse *Conus* venom samples, with the main aim to predict and identify potentially interesting conopeptides in an efficient way. Some bioinformatics techniques have been applied to predict and design novel conopeptide sequences, related targets, and their binding modes. This review provides an overview of current knowledge on the high diversity of conopeptides and multiomics advances in high-throughput prediction of novel conopeptide sequences, as well as molecular modeling and design of potential drugs based on the predicted or validated interactions between these toxins and their molecular targets.

## 1. Introduction

As disulfide-rich small peptides, conopeptides (also known as conotoxins) have been proved to be valuable pharmaceutical tools that target various voltage-gated and ligand-gated ion channels, neurotransmitter transporters, and receptors in the central and peripheral nervous systems [1–3]. They are often ideal resources for development of new drugs to play important roles in neurobiological research [4]. Over the past few decades, they have been used for treatment of many human diseases such as chronic pain, Parkinson’s disease, Alzheimer’s disease, epilepsy, diabetes, and cancer [5–10]. The most famous *ω*-MVIIA, commercially named as Ziconotide, was approved by the US Food and Drug Administration (FDA) in 2004 to treat chronic pain [11]. In addition, some conopeptides have been validated with a high insecticidal activity [12, 13], potentially becoming powerful pesticides when further developed with recombinant baculovirus or to be insect-resistant crop varieties.

Given that there are up to 800 species of cone snails in the world, and each *Conus* species has more than 1,000 conopeptides, it is reasonable to reach a temporary estimate of over 800,000 candidate conopeptides [14]. However, only a total of 8,455 conopeptide sequences have been deposited in the Universal Protein Resource (UniProt; April 3, 2022). Moreover, functions of most conopeptides are not well known. In recent decades, with the rapid development of omics technologies and bioinformatics tools, more and more novel *Conus* venom peptides have been predicted and validated [14, 15]. In the face of such a big number, and the high cost and long period of experimental validations, we fall into an embarrassed condition with accumulative difficulty to resolve conopeptide functions based on biochemical experiments. Recent development of high-throughput computer-assisted methods provides a good opportunity to identify conopeptide families and understand their functions in a more efficient and accurate way, avoiding many shortcomings of those traditional methods [16, 17]. Therefore, research and development of advanced bioinformatics techniques are becoming very important for efficient identification of conopeptide sequences, as well as prediction of sterostructures and potential targets on the basis of sequence alignments [18]. Since the numerous data accumulated by multiomics sequencing support diverse conopeptides as putative analgesics, addiction therapies, and insecticides [3], we can fulfill high-throughput prediction and design of novel conopeptides for in-depth biomedical research and drug development.

This review paper aims to provide an overview of current knowledge on the high diversity of conopeptides in diverse *Conus* species and recent advances in multiomics projects for high-throughput prediction of novel conopeptide sequences, as well as molecular modeling and design of potential drugs based on the predicted and validated interactions between these peptides and their molecular targets.

## 2. High Diversity of Cone Snails for Production of Numerous Conotoxins

**Figure 1:**
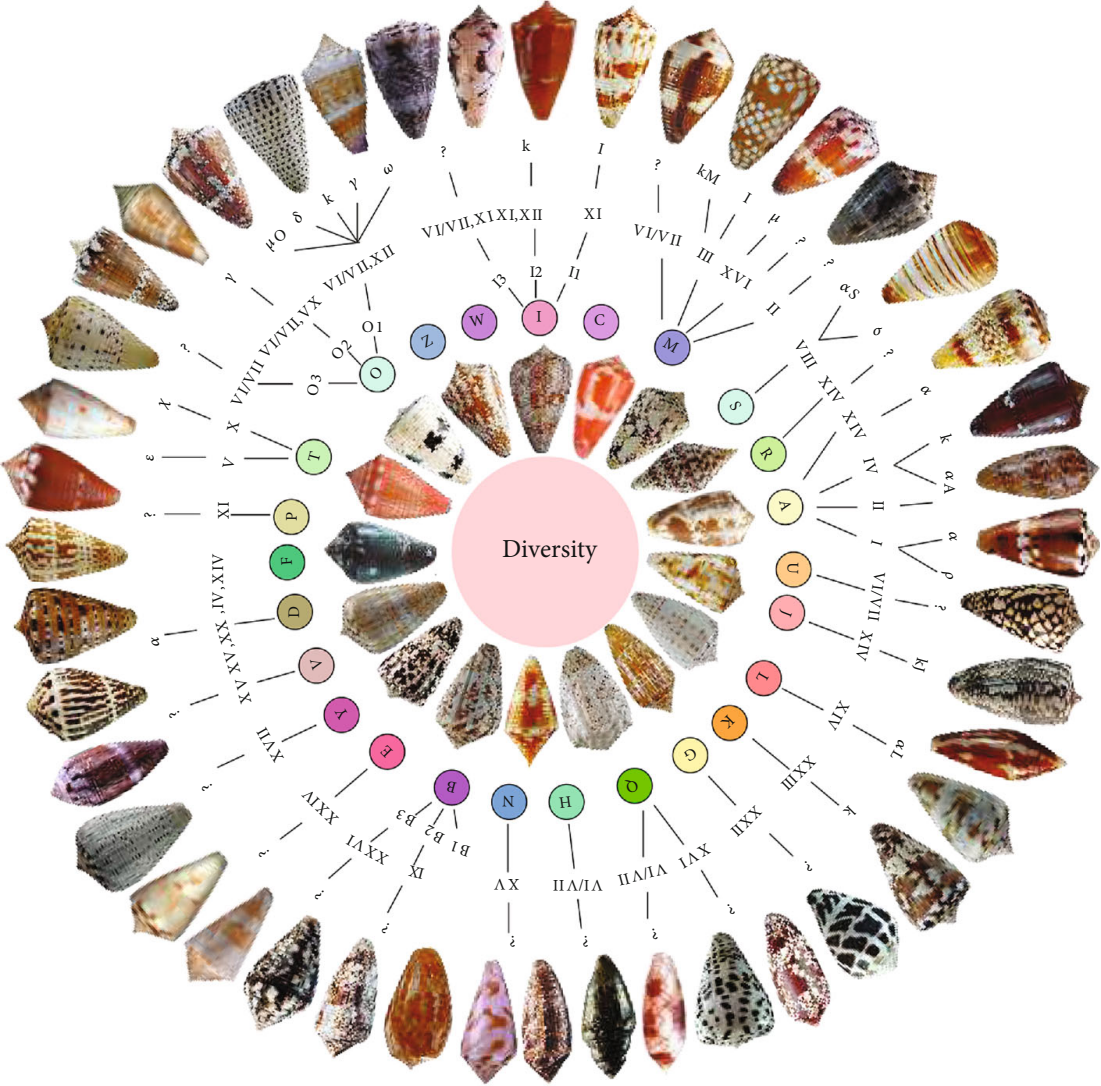
High diversity of cone snails and their conopeptides.

The genus *Conus* belongs to one of the most diverse and taxonomically complex superfamily Conoidea [19]. According to many recent estimations, there are around 800 *Conus* species, and most of them are still not well characterized [20, 21]. Cone snails have long been the interest of worldwide collectors because of their beautiful shell patterns (Figure 1), while identification of various *Conus* species is mainly based on the shapes and colors of their shells. However, due to regional and intra-specific variations, it is very difficult to practically determine the accurate species of living cone snails only by relying on shell characters [22]. In addition to the beautiful and complex shells, rich conopeptides (see an example in Figure 1) and dietary breadth, along with corresponding taxonomy, population genetics, evolutionary biology, and phylogenetics, have aroused the interest of international scientists and businessmen [23–25].

At the present time, mitochondrial genome (mitogenome) sequencing has become one efficient tool in gastropod taxonomy [26]. These mitogenome sequences present high diversity of gene and nucleotide orders, providing a good mode for examination of detailed pattern, rate, and mechanism of mitogenomic rearrangements, and construction of a precise phylogenetic topology for arrangement comparisons [27]. Due to *Conus* species differentially prefer hunting fishes, worms, or molluscs, they are traditionally classified into piscivorous, vermivorous, or molluscivorous groups, respectively, although sometimes certain cone snails may feed on more than one prey type [28–30]. Accumulated evidence suggests that the common ancestor of diverse cone snails was likely vermivorous, feeding on environmentally available worms. Gao et al. reconstructed a simplified phylogenetic tree of various cone snail species based on their complete mitogenome sequences, confirming this evolutionary tendency in feeding behavior [23]. Meanwhile, it has been generally recognized that venom compositions in various *Conus* species may be adaptively shaped by prey type and dietary breadth [3, 23]. Therefore, the prey taxonomic classification can be predicted by *Conus* venom components, although practical performances were poorly validated [31]. In general, selective pressures driven by diets are considered the major contributor to shape the evolution of *Conus* venoms.

In order to overcome their slow actions and inability to capture fast-moving preys, cone snails have developed a highly sophisticated venomous apparatus, composed of a long venom duct and a goosing venom bulb, for the synthesis, storage, and delivery of functional conopeptides [32, 33]. So far, over 8,000 mature conopeptide sequences are publicly available. With continuous reduction of the cost for transcriptome and proteome sequencing, this number has been increasing rapidly [3, 34–36]. These conopeptides were initially classified into different superfamilies with the assistance of conserved signal sequences and unique cysteine frameworks [3]. Currently, a total classification of 31 superfamilies has been established [37–39]. According to the arrangement of cysteine frameworks, every superfamily can be subdivided into several families. For example, A-superfamily conopeptides, with four representative cysteine frameworks (namely, I, II, IV, and XIV; Figure 1), are therefore subdivided into *α*, *α*A, and *κ*A families; M-superfamily conopeptides, with five cysteine frameworks of II, XIV, III, VI, and VII, are further subdivided into *μ* and *ψ* families; O-superfamily conopeptides have four cysteine frameworks (XII, XV, VI, and VII) and are subsequently divided into *δ*, *μ*O, *ω*, *κ*, and *γ* families [40–43]. The high diversity of cone snails and their conopeptides, with an outline of the 29 major conopeptide superfamilies [19, 21, 37, 44], are summarized in Figure 1 for convenient comparison.

## 3. Multiomics for High-Throughput Prediction of Novel Conopeptides

### 3.1. The Representative Database, ConoServer

At present, several popular databases including NCBI Genbank/GenPept [45], UniProt [46], and the Protein Data Bank (PDB) [47] play important roles in simplifying the acquisition of target protein or peptide sequences and related three-dimensional (3D) structures [48]. However, many details of venom toxins, especially the conopeptide nomenclature and related pharmacological activities, are not standardized in these general databases; therefore, mining conopeptides from these public databases is usually difficult. For the convenience of users and readers, some special databases of venomous animals have been established progressively. For instance, ConoServer [49], Arachnoserver [50], and Indigenous Snake Species of Bangladesh (ISOB) [51] include venom and toxin details for various cone snails, spiders, and snakes, respectively.

Here, we introduce the representative ConoServer, a specific and powerful database for conopeptides with an easy and convenient access to related sequences, structural, and activity data [49, 52]. To date (by April 4, 2022), ConoServer has organized a total of 2,986 nucleotide sequences (from 90 *Conus* species), 8,361 protein sequences (from 122 *Conus* species), and 229 3D structures (from 48 *Conus* species). This useful database offers a comprehensive overview of various conopeptides. More importantly, it also provides comparable relationships among sequence, structure, and activity, which is particularly instructive for practical drug design [53, 54].

**Figure 2:**
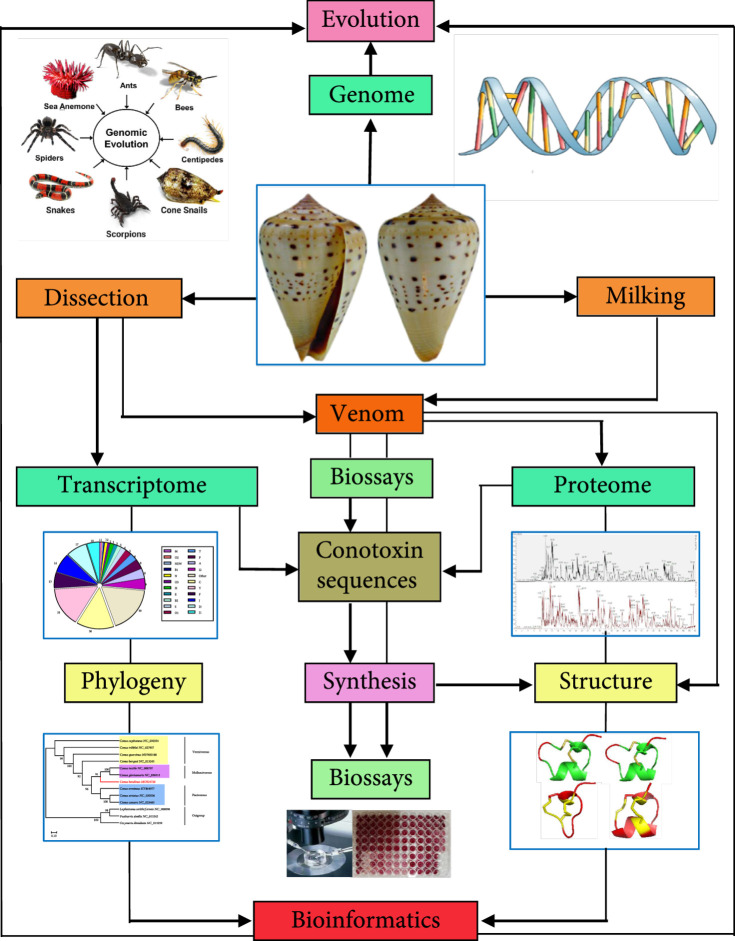
Integration of proteomics, transcriptomics, genomics, and bioinformatics to study cone snail venoms. This integrative strategy has been applied to excavate numerous conopeptide sequences.

To overcome the limitations of those traditional low-efficient biodiscovery methods, advanced multiomics approaches including transcriptome, proteome, and genome sequencing have been introduced to accelerate the identification and characterization of numerous conopeptides from various cone snails [14] (Figure 2). These big-data-based methods are integrated to reveal the detailed complexity of *Conus* venoms and to provide novel insights into their biology and evolution [55]. They have been employed to generate a lot of new conopeptide sequences [39, 56]. The main challenge for these studies, however, is the effective combination of multiomics datasets and confirmatory pharmacological results. Recently, two bioinformatics tools have been established in ConoServer to customize the identification and characterization of new conopeptide sequences [49, 57, 58]. ConoPrec supports an analysis of conopeptide precursor sequences predicted by transcriptome sequencing, while ConoMass is used to match conopeptide masses predicted from transcripts with a list of masses obtained experimentally by proteome sequencing of *Conus* venoms [28, 49]. Therefore, the integrated ConoServer database can remarkably improve the validation of integrative venomics data to accelerate the discovery of more and more novel conopeptide sequences.

### 3.2. Transcriptomic Analyses of Conopeptides from Cone Snails

The first *Conus* transcriptome was reported in 2011 by Olivera and colleagues at University of Utah, although only 30 putative conopeptide sequences were identified in this pioneering study [59]. This primary work revealed for the first time that conopeptides are mainly transcribed within the venom duct of the representative *C. bullatus* and more importantly provided a detailed pipeline for efficient prediction of putative conopeptides.

Total conopeptide transcripts, varying from tens to 522 [39] or more (Table 1), can be identified from various *Conus* species by transcriptome sequencing [22, 59, 60, 62–65]. Diverse samples, including individual or pooled snail(s) and single or pooled tissue(s), have been examined (Table 1) for practical projects in various countries, especially in the USA, Australia, and China. Usually, extraction of total RNA, isolation of mRNAs, and library construction are routinely performed before sequencing and data processing [59].

**Table 1:** Transcriptome sequencing of representative *Conus* species.

	Examined species	Sample diversity	No. of identified Conopeptide transcripts	Reference
V1${}^{*}$	*C. betulinus*	Venom duct, venom bulb	215	[59]
V2	*C. quercinus*	Venom duct, venom bulb, salivary gland	133	[60]
V3	*C. Caracteristicus*,	Venom duct	48-118	[22]
	*C. generalis*,			
	*C. quercinus*			
M1${}^{*}$	*C. Episcopatus*	Venom duct, radular sac, salivary gland	305	[61]
M2	*C. Ammiralis*	Venom duct	242	[62]
M3	*C. marmoreus*	Venom duct	264	[63]
P1${}^{*}$	*C. tulipa*	Venom duct	328-522	[39]
P2	*C. consors*	Venom duct	53	[64]
P3	*C. bullatus*	Venom duct	410	[65]

${}^{*}$: V = vermivorous; M = molluscivorous; P = piscivorous.

A local reference database of known conopeptide sequences can be established [59] from the ConoServer [49] and previously published papers for prediction of putative conopeptide sequences in any assembled transcriptome data. Early examination using the ConoPrec tool [49] and manual removal of any transcript with duplication or truncated mature region are required before generation of the final list. Transcription of each unique conopeptide gene is often calculated using the representative parameter RPKM (reads per kilobase of transcript per million mapped reads) [66] for comparisons.

**Figure 3:**
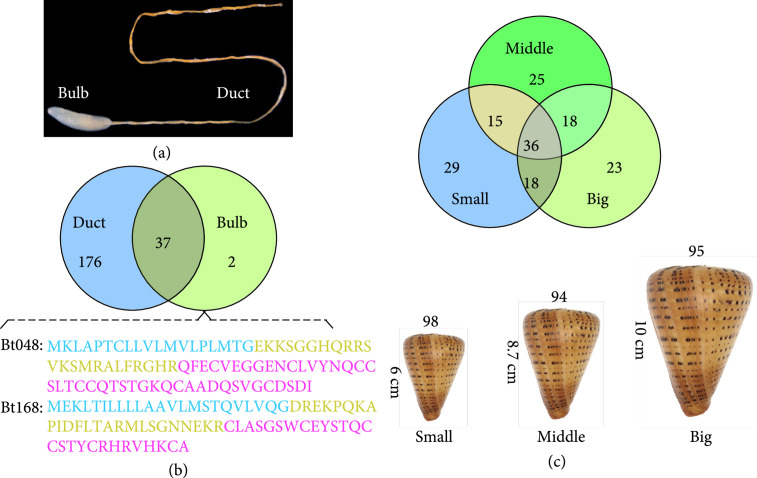
Tissue-specific distribution and developmental changes of conopeptide transcripts in *C. betulinus* (modified from [59]). (a) Dissection of the venom duct and bulb. (b) Tissue-specific distribution of conopeptide genes from the pooled datasets. The blue, yellow, and pink sequences represent the signal peptide, pro region, and mature peptide, respectively. (c) Developmental changes of conopeptide transcripts (total numbers in the below panel [59]). Top panel shows the overlaps among three different body-sized (6-10 cm in length) specimens.

For the reported *Conus* transcriptome works, researchers often focus on tissue-specific distribution and/or developmental changes of predicted conopeptide transcripts. For example, a total of 215 conopeptide transcripts (Bt001–Bt215; Figure 3) were obtained after sequencing of various samples from *C. betulinus* (the most popular *Conus* resident of the South China Sea), including cDNA libraries (46), and mRNAs from normalized venom ducts (123), non-normalized Small (98)/Middle (94)/Big (95) venom ducts, and non-normalized venom bulb (39) [59]. Each of them has at least one amino acid variance in the sequence of mature region. Among them, 178 conopeptides were sorted into 20 known superfamilies [3, 59].

Differential transcription of various conopeptides in both venom duct and venom bulb (Figure 3(a)) was revealed by transcriptome sequencing and validated in part by quantitative RT-PCR [59]. As expected, the total number of unique conopeptide transcripts in the latter (39) was less than half of that in the former (94), since venom duct is the main organ for conopeptide synthesis [3, 65]. Between the two transcriptomes of middle-sized snails, 28 conopeptide transcripts are shared, whereas 66 (for the venom duct) and 11 (for the venom bulb) are unique. When the bulb dataset was further aligned against the combined venom duct datasets, two transcripts (Bt048 and Bt168; Figure 3(b)) are still identified to be unique in the venom bulb [59]. Although the potential cross contamination cannot be eliminated (due to the difficulty to remove the tiny partial duct inside the connective venom bulb [59]; see more details in Figure 3(a)), these data shed lights on the presence of conopeptides in the venom duct, a minor contributor of the toxin resource.

Peng et al. also examined developmental changes in total number (Figure 3(c)) and transcription levels of various conopeptide transcripts [59]. A total of 36 are shared among different body-sized specimens (see detailed classification of Big, Middle. and Small datasets in the below panel of Figure 3(c)), and around 50 are shared in each pairing (Small and Middle, Middle and Big, and Big and Small; top panel in Figure 3(c)). The top 20 conopeptide transcripts with the highest RPKM values from each of the three datasets were selected for comparison. In general, their transcription levels in the Middle specimens are often higher than those in the other two groups. Interestingly, eight are shared among the top 20s of the three datasets, although their RPKM rankings are variable [59]. In the coming future, it is obviously reliable to obtain more novel conopeptide sequences via transcriptome sequencing of more tissues apart from the traditional venom duct and more samples at different developmental stages.

### 3.3. Proteomic Analyses of Conopeptides from Cone Snails

In early studies, venoms were popularly sequenced by mass spectrometry (MS), but this technology is difficult to be extensively applied due to its sample-consuming, time-consuming, and low-throughput characters [59, 60]. With recent development of advanced MS systems and powerful bioinformatics tools, proteomics technology has become an effective and high-throughput strategy to identify lots of novel conopeptide sequences [67]. MS allows additional characterization of post-translational modifications (PTMs), which cannot be revealed by other methods including genome and transcriptome sequencing. In recent decades, MS-based proteomics has been used to explore venom components of various cone snails.

For instance, advanced TripleTOF 5600 and Q Exactive HF were employed to perform proteomics studies for exploration of various conopeptides from *C. betulinus* [68]. Using the 215 previously identified conopeptide sequences [59] as the reference, a total of 1,522 conopeptide amino acid sequences, corresponding to 121 transcripts, were detected by the Q Exactive HF system (without PTM details). Similarly, 773 conopeptide sequences, matching 92 known conopeptide transcripts, were characterized by the TripleTOF system without consideration of any PTMs. However, only 31.8% (282) of identified conopeptides, covering 77.2% (71) transcripts, were shared between the two MS datasets [68].

Proteomics has also been employed for the discovery of *Conus* switching of variable venom compositions, when they are at a prey or defend condition. In piscivorous *C. striatus*, milked defensive venom was full of inhibitory *α*-, *ω*-, and *μ*-conopeptides in comparison with a milked predatory venom [69, 70]. However, in addition to the sharp increase of conopeptides observed in *Conus* venoms, other components including various enzymes and hormones were also found. Interestingly, insulin analogs are identified in *Conus* venoms. There are 34 insulins from ten representative *Conus* species, exhibiting diverse conoinsulin sequences for potential activation on various animal (including human) insulin receptors [71–73].

### 3.4. Genomic Analyses of Conotoxins from Cone Snails

Over ten years ago, an early attempt to sequence *C. bullatus* genome was published [65]. It is a trail of genome survey with numerous fragments, while many properties were characterized in detail, such as overall GC content and estimated genome size. A partial genome assembly was generated five years later for *C. tribblei* [74]. More recently, Phuong and Mahardika successfully recovered exon sequences of conopeptide gene superfamilies from 32 *Conus* venom genomes [75], with assistance of targeted sequencing techniques. A set of unpublished genome data for *C. consors* by a different research team were also deposited at NCBI (GCA_004193615.1) for public availability and comparison.

**Figure 4:**
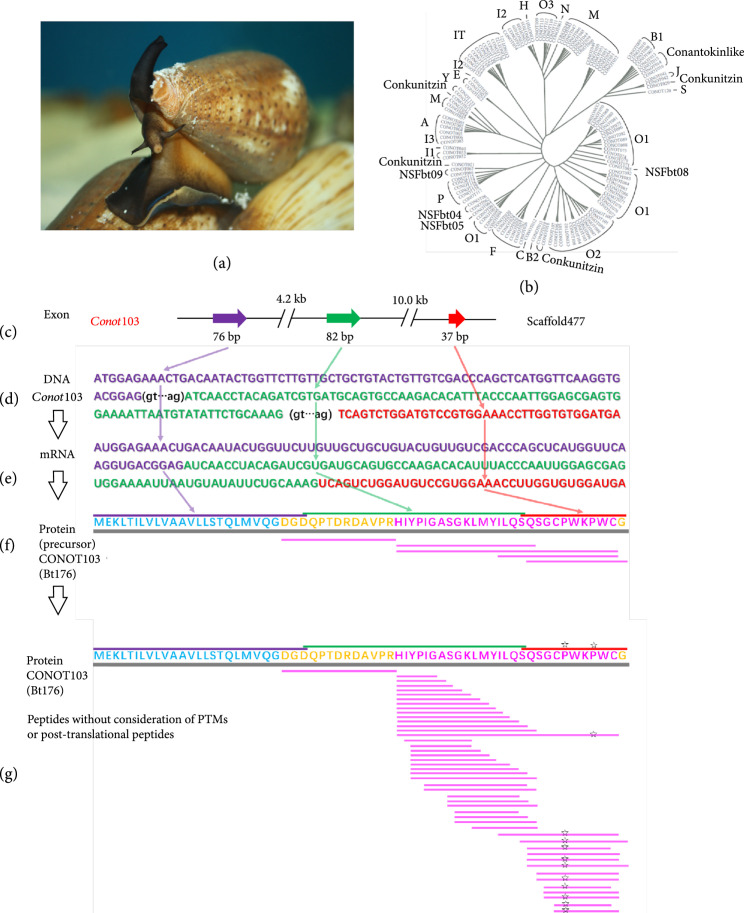
Identified conopeptide transcripts and post-transcriptional peptides in *C. betulinus* (modified from [68]). (a) Picture of a cultured *C. betulinus*. (b) Phylogeny of identified conopeptide transcripts [59, 68]. (c–f) Establishment of a primary central dogma for the representative gene *conot103*, which transcribes *Bt176* [59] and translates CONOT103 [68]. The blue, yellow, and pink sequences represent signal peptide, pro region, and mature peptide, respectively. (g) MS detection of functional conopeptides.

In the February of 2021, the first *Conus* genome assembly was reported by Peng et al. in *Cell Discovery* [68], which combined short sequencing reads (Illumina) and long reads (PacBio). After integration of genome, transcriptome, and peptidome data in the same *C. betulinus* (Figure 4(a)), the authors predicted a primary genetic central dogma for 133 identified conopeptide genes (named as *conot001*–*conot133*; Figure 4(b)), which are genome-widely distributed with Hi-C assembled evidence. A rough number ratio of ~1 : 1 : 1 : 10s was established for the total genes : transcripts : proteins : post-translationally modified peptides (Figures 4(c)–4(g)); however, it may be special in this vermivorous species, since there are accumulated reports of various *Conus* genome sizes along with big number ranges of conopeptide genes (120-859 [75]), transcripts (100-522 [39] or more), and peptides [68] in diverse *Conus* species. Interestingly, in individual *C. betulinus*, only 61 (45.9%) conopeptide genes were proved to be transcribed with transcriptomic evidence [68], which is covered by the previously reported transcription ranges of 24~63% in 32 *Conus* species [75].

Peng et al. also obtained peptide processing details at the protein level [68], predicting a big number of venom conopeptides with variable alternative cleavage sites (the most of 15 in this study), post-translational modifications, and N- or C-terminal partial truncations [75], can illuminate how the low sum of 133 genes and~123 transcripts can produce several thousands of conopeptides in individual venom of *C. betulinus*. Taking the various modifications of identified conopeptides into consideration, it is reasonable to estimate that the maximal sequence number of functional conopeptides in *C. betulinus* may reach up to 6,653 [68], which would be verified by more sensitive and advanced MS systems.

Three months later (May of 2021), a group of European scientists published another *Conus* genome assembly [76]. The assembled genome of their examined Mediterranean *Conus* (*Lautoconus ventricosus*) is 3.59 Gb, similar in size to the assembly of 3.43 Gb for *C. betulinus* [68]. Although only partial genome sequences (less than 50%) for both *Conus* species were constructed into 35 fragmented pseudochromosomes [68, 76], we are positively waiting for development of advanced sequencing techniques and novel bioinformatics packages to obtain high-quality chromosome-level genome assemblies for various *Conus* species.

## 4. High-Throughput Design of Novel Conopeptides for Biomedical Research and Drug Development

A growing availability of computer-aided drug design methods has been witnessed in recent years, which can be also used for high-throughput design of novel conopeptide sequences for biomedical research and drug development [18, 77–79]. Machine learning methods are useful for prediction of conopeptide sterostructures and functions with high accuracy on the basis of amino acid sequences. Docking studies reveal that conopeptides selectively bind to their receptors. Molecular dynamics (MD) accurately evaluate binding pathways and dynamical conformations. Integrated computational approaches are being employed to identify and optimize conopeptides for specific applications [18, 78, 79].

### 4.1. Machine Learning for Prediction, Screening, and De Novo Design of Novel Conopeptides

With identification of more and more new conopeptide sequences by means of transcriptomics and proteomics, it is important to define their functions for biomedical applications. As mentioned above, candidate conopeptide genes can be online aligned to similar sequences with previously known functions. Online operations are available under the public Uniprot, ConoServer, ConoPrec, ConoDictor, and Conosorter [49, 80]. Extensive works can be fulfilled to predict biological roles for any novel conopeptide by construction of machine learning algorithms to find its target receptors. For example, ICTCPred machine learning algorithms have demonstrated good performance in identification of novel conopeptides that target voltage-gated channels, achieving predictive accuracies of about 90% [81, 82]. However, these techniques can only identify novel sequences with high similarity to previously known conopeptides. To overcome these difficulties for prediction, ConusPipe, a novel machine learning tool, was developed to detect potential mRNA sequences within sequenced *Conus* transcriptomes but without conopeptide homologs [17]. Despite availability of many algorithms for accurate prediction of novel conopeptides with potential target receptors, these data do not allow illumination of detailed mechanisms for their selective binding to the target receptors. Such mechanisms can be elucidated through other methods, such as modeling docking and MD analysis.

Machine learning has also been successfully employed to develop novel de novo design approaches or other small peptides and large proteins. Many new algorithms and models have been constructed [83, 84], and some have been successfully applied for design of other active peptides such as antimicrobial peptides [85, 86]. However, few studies have focused on the design of conopeptides. A previous study reported the design of a *μ*-conotoxin KIIIA peptidomimetic, although machine learning was not mentioned [87]. The major challenge in *de novo* design of chemicals is the synthetic accessibility of deduced molecular structures [88], especially for those long and complicated proteins. However, machine learning-enabled *de novo* design of functional compounds including conotoxins will definitely prompt the process of drug development.

### 4.2. Modeling Docking and MD Analysis for Prediction of the Binding Structures and Dynamical Conformations

Modeling docking can predict the structures of conopeptide complex with receptors and their relative affinity, which may help to evaluate the library of conopeptide sequences for possibility of binding to the target proteins [18, 78]. Docking procedure includes searching the success “docked” complex through the conformational space, followed by scoring (the hit rate) to list the binding probability of conopeptides to the protein targets [78, 89]. The “lock-and-key” mechanism can explain how these conopeptides select different receptors and how different structures determine diverse functions. Docking is concerned with the conformational space of the target proteins (lock) and orientation of conopeptides (key). Docking usually relies on available crystal or cryo-electron microscopy macromolecular structures to determine those conopeptides that bind to the protein targets with detectable affinity. Related criteria to define the success “docked” scoring may be subjective, and the success “docked” is usually used to validate only those hit complexes with confirmation by crystal or cryo-electron microscopy structures [89].

Docking studies are always in company with MD simulations. MD protocols can complement and develop docking for prediction of molecular mechanisms by providing atomistic details of “hand and glove-like” association events. MD simulation methods are most adequate for characterization of flexible binding sites and accurate evaluation of binding pathways, kinetics, and thermodynamics. They are used frequently to guide further optimization of structure discovery in docking studies. Even docking has identified conopeptide interactions with particular targets, and MD will provide insights in detailed characterization and prediction of which residue interactions are stable and contribute mostly to binding [90, 91].

**Figure 5:**
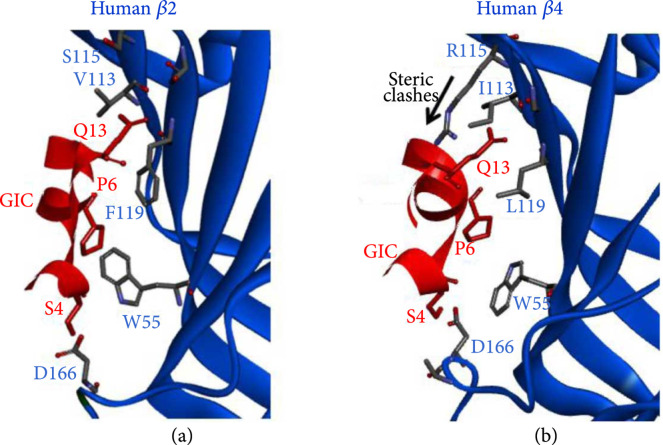
Computational approaches for high-throughput design and identification of novel conopeptides. (a) Computer modeling for identification of conopeptide GIC with high selectivity of human *α*3*β*2 vs *α*3*β*4 nAChR. GIC exhibits a high affinity to the human *α*3*β*2 nAChR, which is 700 times higher than the human *α*3*β*4 nAChR. Although *α*3*β*2 and *α*3*β*4 have the same *α*3 subunit, the interesting difference is caused by the complementary subunit *β*2 and *β*4. Computer modeling indicates that the pocket in human *β*2 is able to accommodate the GIC without steric clashes. (b) Arg-115 in the pocket of human *β*4 brings steric clashes with the GIC and then disfavors GIC binding [84].

**Figure 6:**
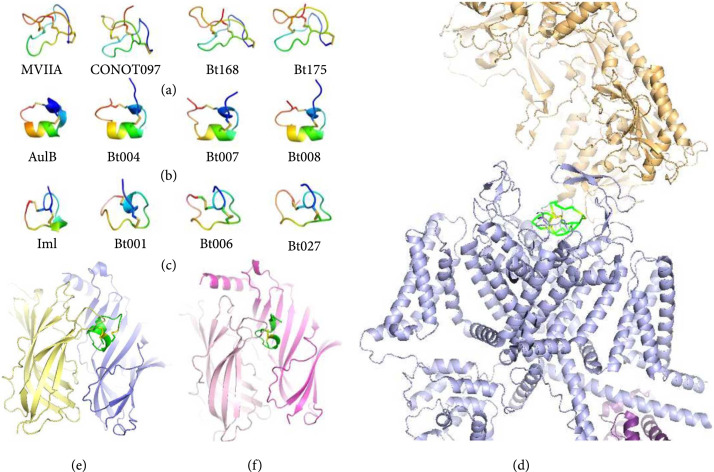
Predicted homologous conopeptides based on the 3D structures of MVIIA, AuIB, ImI, and their receptors [79]. Homologous conopeptides of (a) MVIIA, (b) AuIB, and (c) ImI are predicted for comparisons. Top three best hits from homologous alignments and models of these conopeptide sequences were generated using MODELLER (version 9v12) with MVIIA (PDB: 1OMG), AuIB (PDB: 1MXN), and ImI (PDB: 2BC7) as corresponding templates. Prediction of homologous conopeptides with their receptors relies on the 3D structures of (d) MVIIA complexed with Cav2.2 calcium channel (PDB: 7MIX) [95], (e) AuIB complexed with *α*3*β*2 nAChR, and (f) ImI complexed with *α*7 nAChR, respectively.

With the development of nuclear magnetic resonance (NMR), X-ray crystallography, and cryo-electron microscopy, 3D structures of many conopeptides and their receptors are available to benefit docking and molecular dynamics. Based on the structures of these conopeptide complexes with corresponding receptors, computer models further reveal selectivity of certain conopeptide residues for different protein targets and discovery of potent analogs. For example, based on the crystal structure of the *α*-conopeptide GIC in complex with Ac-AchBP, Peng et al. [68] applied docking and MD to extend these data from the X-ray structures of the AChBP complexes to all nAChR subtypes and to elucidate the mechanism of GIC’s high selectivity for human *α*3*β*2 nicotinic acetylcholine receptor (nAChR; Figure 5) [92]. Meanwhile, depending on the 3D structures of three representative conopeptides (MVIIA, AuIB, and ImI) and their receptors, a homologous modeling method was employed to predict sterostructures of many interesting homologous counterparts (Figure 6). These findings imply that novel conopeptide sequences may be developed as valuable drug-related analgesics, addiction therapies, and insecticides, respectively [79]. Similarly, based on a conopeptide bond to nAChR homology structures, new conopeptides were discovered through docking and virtual screening by an algorithm called ToxDock [93]. Usually, development of new drugs depends on animal models to test, but human receptors remarkably different from their animal counterparts. Docking and MD can work together to provide insights in the important residues for binding to human receptors, thereby aiding to design of potential conopeptide analogs for in-depth clinical trials [93, 94].

### 4.3. Integrated Approaches for Rational Design of Conopeptides with Specific Applications

Computational algorithms have mainly been applied in two fields for conopeptide design, including conopeptide identification and optimization. For the identification, machine learning and docking methods are chosen to screen large libraries of conopeptide molecules by prediction of binding to target proteins. For the conopeptide optimization, MD-based protocols are a good choice of calculating different residue-free energies and binding kinetics for discovery of more potent analogs. Integrating the two approaches can support high-throughput studies to classify diverse conopeptides [78, 96].

In addition to prediction and design, functional validation is also rather important and only become the rate-limiting step for peptide studies. It is therefore required to integrate validation experiments with computational predictions so as to improve the accuracy of in-depth experiments. Over the past decade, a series of innovative integrating approaches have been introduced. For example, Peng et al. [59] performed venom transcriptome sequencing of *C. betulinus* and constructed a big library of 215 conopeptide transcripts with solid evidence. Among them, six sequences similar to *α*-ImI were identified by integrating with homology search, and two sequences were proved with desirable insecticidal properties by combining with bioactive experiments [13]. Younis and Rashid integrated docking and MD simulations to characterize several conopeptides as potential anticancer drugs; among them, *α*-BuIA strongly binds to the lysophosphatidic acid receptor 6 (LPAR6) for involvement in several aggressive cancers [97]. Mansbach et al. applied a graph-directed approach by combining structure modeling and experimental guidance to obtain a large library of conopeptide structures for high-throughput screening and also to supply a big set of ranked sequences for in-depth structure validation with experiments [98].

### 4.4. Protein Folding Shape Codes

More than a decade ago, Yang developed a novel bioinformatics package, termed as Protein Folding Shape Codes (PFSCs) [99], which can be used to describe comprehensive confirmations for various conopeptide sequences [3].

**Figure 7:**
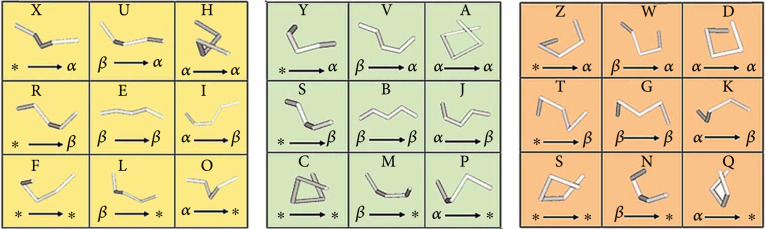
Diagram of the 27 PFSC vectors (adopted from Yang’s paper [99] with permission).

**Figure 8:**
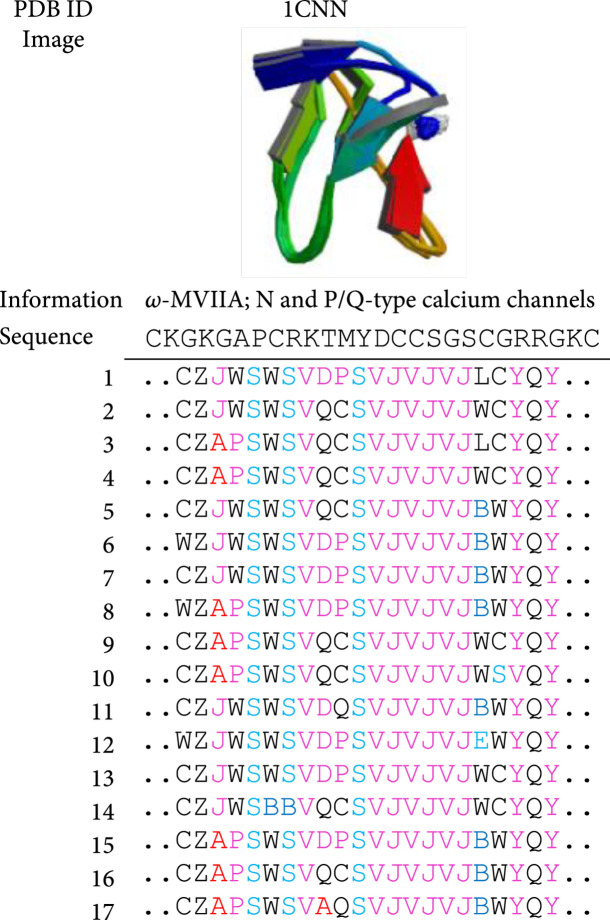
Conformation descriptions of the representative *ω*-MVIIA (modified from [3]). Section A lists the details of 17 confirmations.

For a high-quality shape description, the PFSC method integrates variable geometric, morphological, and 3D topological characters as a visible algorithmic process [99]. Represented by the 26 alphabet letters (from A to Z) and the “$” sign, an interesting set of 27 PFSC vectors can cover the complete folding shapes of any five successive amino acids (Figure 7), generating an instructive description for potential 3D structures. These vectors represent 27 different folding shapes, and every vector is represented by a collection of a letter, a folding shape pattern, and an arrow (see more details in the three blocks of Figure 8). Those “*α*,” “*β*,” or “∗” represent different folding features, corresponding to *α*-helix, *β*-strand, or random coil, respectively [99].

Consequently, any conformation of a conopeptide sequence can be established by a string of letters as a meaningful protein structure fingerprint, which can be used to compare similarity and difference with other conopeptide(s) [3]. Here, the most well-known *ω*-MVIIA [100] is taken as an example. As we know, folding changes are usually difficult to present in relation to a known 3D structure; however, it will be easy to demonstrate the fine variances of folding shapes among multiple isomers when using the 27 PFSC vectors to generate protein structure fingerprints [3, 99]. Section A in Figure 8 summarizes the possible conformations by PFSCs in the order of possibility (from high to low), according to the given 3D structural image (PDB ID: 1CNN) [3]. The binding sites for this conopeptide sequence to any target receptor can also be illuminated by using the 27 PFSCs [93]; therefore, a better understanding of variable and diverse interactions between it and its receptors, such as nAChRs and N or P/Q-type voltage-dependent calcium channels, can be achieved at the same time.

Protein and peptide conformation analysis is very important due to its prediction of potential folding structures at different conditions and identification of possible misfoldings that may be related to clinical human diseases [99]. These PFSCs are powerful for analyzing protein confirmations, with detailed exhibition of local structural folding features. In summary, the 27 PFSC vectors (Figure 7) are good images for generation of abundant details for the conopeptide conformations, with assistance of a known 3D structure. On the other hand, they can predict lots of folding variations to establish conformations for any conopeptide sequence without available 3D structures, which may help to design novel conopeptide sequences. They may also provide an alternative tool to explore the detailed mechanisms of interactions between various conopeptides and important function-related target proteins, which are instructive for development of interesting conopeptides as practical marine drugs.

## 5. Perspectives

Cone snail venoms have been considered a treasure library of small bioactive peptides, which can be used for development of novel marine drugs and lead compounds [101]. To date, about 8,000 mature conopeptide sequences have been discovered from various *Conus* venoms, but only a few have been characterized with detailed structures and functions [102, 103]. Due to the explosive growth of big datasets from transcriptome, proteome, and genome sequencing, traditional experiment-based methods can no longer meet the numerous needs of rapid prediction of conopeptide sequences, 3D structures, and target proteins [50, 104, 105].

Computer-assisted high-throughput prediction and design technologies play a crucial role in conopeptide discovery and development. Novel machine learning, deep learning algorithms, and visualization techniques have been introduced to facilitate conopeptide exploration. Although at present there are some inevitable obstacles in the practical process of conopeptide discovery and drug development, and a lot of more investigations are required to integrate computing tools into the drug discovery process, we are convinced that computing technologies along with multiomics data will bring revolutionary changes to the worldwide drug discovery and development in the near future [106–108]. In summary, high-throughput prediction and design technologies including artificial intelligence and deep learning advancements are providing a good opportunity for rational process of marine drug design and discovery from various predicted and/or validated conopeptides. These powerful strategies and tools can be generally applicable to predict and design of other peptides and proteins, which will eventually generate a profound impact on mankind knowledge and health.
